# Improving Maternal and Newborn Health: Effectiveness of a Community Health Worker Program in Rural Kenya

**DOI:** 10.1371/journal.pone.0104027

**Published:** 2014-08-04

**Authors:** Mary B. Adam, Maria Dillmann, Mei-kuang Chen, Simon Mbugua, Joram Ndung’u, Priscilla Mumbi, Eunice Waweru, Peter Meissner

**Affiliations:** 1 AIC Kijabe Medical Center, Kijabe, Kenya; 2 Department of Pediatric and Adolescent Medicine, Ulm University, Ulm, Germany; 3 University of Arizona, Tucson, Arizona, United States of America; Indiana University and Moi University, United States of America

## Abstract

**Background:**

Volunteer community health workers (CHWs) form an important element of many health systems, and in Kenya these volunteers are the foundation for promoting behavior change through health education, earlier case identification, and timely referral to trained health care providers. This study examines the effectiveness of a community health worker project conducted in rural Kenya that sought to promote improved knowledge of maternal newborn health and to increase deliveries under skilled attendance.

**Methods:**

The study utilized a quasi-experimental nonequivalent design that examined relevant demographic items and knowledge about maternal and newborn health combined with a comprehensive retrospective birth history of women’s children using oral interviews of women who were exposed to health messages delivered by CHWs and those who were not exposed. The project trained CHWs in three geographically distinct areas.

**Results:**

Mean knowledge scores were higher in those women who reported being exposed to the health messages from CHWs, Eburru 32.3 versus 29.2, Kinale 21.8 vs 20.7, Nyakio 26.6 vs 23.8. The number of women delivering under skilled attendance was higher for those mothers who reported exposure to one or more health messages, compared to those who did not. The percentage of facility deliveries for women exposed to health messages by CHWs versus non-exposed was: Eburru 46% versus 19%; Kinale 94% versus 73%: and Nyakio 80% versus 78%.

**Conclusion:**

The delivery of health messages by CHWs increased knowledge of maternal and newborn care among women in the local community and encouraged deliveries under skilled attendance.

## Introduction

Improving maternal, newborn, and child health is an important priority in sub-Saharan Africa where most births and deaths occur at home. Scientific evidence for effective low cost interventions to reduce the rate of maternal and newborn deaths continues to accumulate [1]–[8]. Reducing home deliveries or deliveries with an unskilled birth attendant combined with earlier identification of danger signs in a mother or newborn form an indispensable part of many of these evidenced based interventions. Community Health Workers (CHWs), a broad category of non-professional health workers is often the first point of contact in these interventions and provides an essential link to clinical services. This study examines the effectiveness of a CHW Project conducted in rural Kenya that sought to promote improved knowledge of maternal and newborn health and to increase deliveries under skilled attendance.

Kenya, like many countries in sub-Saharan Africa, suffers from a critical shortage of health care workers. Kenya has responded to the shortage by developing The Kenya Community Strategy for Health [9], a strategy that utilizes lay volunteers CHWs as the foundation for promoting behavior change through health education, earlier case identification, and timely referral to trained health care providers. In choosing this approach Kenya builds on evidence that task sharing is both possible and effective in promoting health behaviors at the community level [10]–[12]. Fifty-six % of Kenyan women deliver without the help of a skilled birth attendant [13], and many communities have not yet had the benefit that could come from trained CHWs.

CHWs play a key role in providing the motivation for women to change behaviors surrounding birth, delivery, and newborn care; behaviors deeply rooted in a specific social-cultural context. CHWs form an entry point into multiple social networks, networks that are essential in order to build the requisite trust and momentum for any type of change in health behaviors [14].

That is why influential local community members are recruited. However, retaining volunteers remains a challenge [15], [16]. Identifying strategies that successfully promote healthier behavior using the Kenyan scope of CHW activities combined with strategies to retain volunteers is needed as the country scales up its Community Strategy for Health.

In this paper we describe the effectiveness of a volunteer community health worker project that utilized a health prevention and promotion role for CHWs consistent with the Kenya national strategy; utilizing the package of evidenced based interventions Kenya has chosen to improve country performance on Millennium Development Goals 4 & 5 on maternal and neonatal health. The primary aim of the study was to test the effectiveness of using CHWs to improve the local communities’ knowledge of maternal and newborn care and secondarily to examine the effectiveness of this strategy in promoting delivery with a skilled birth attendant.

## Methods

Ethics Statement: Prior to beginning the research study, letters of research collaboration and support were received from all District Health Management Teams and all area chiefs. Human subjects approval was obtained from the Kijabe Hospital Ethics Committee and from the University of Ulm Ethics Committee in Germany. All study participants gave verbal voluntary informed consent. Most participants in this area spoke a vernacular language that has not been taught in schools for decades. English and Kiswahili were declared national languages with Kenya’s independence. Interviews were done in the vernacular language and consent to participate was documented by the interviewer as they recorded responses.

We recruited 83 volunteers from rural communities in 3 geographic areas representing 3 of Kenya’s 47 counties. People in these regions were primarily subsistence farmers who were not employed in the formal economy. These volunteers were trained in their home area by staff from the Kijabe Community Health Maternal Newborn Project (KCHMN) in the information they would need to know to be effective CHWs. Volunteers began to serve in their local villages by sharing health messages one on one and with small groups as soon as they learned a lesson and were confident they could do the work required. They did not need to complete all sessions of the training to start sharing health messages. Training required mastery of material in six different sessions and each session was followed by mentored skill practice in the ways in which CHWs were to carry out their activities. Each class of 10–15 volunteers demonstrated competence on all the material before the next class started. No stipends were given. This is in contrast to many CHW trainings implemented by NGOs in Kenya where stipends for attendance at training and an ongoing monthly stipend for working in the community are given.

In order to assess the effectiveness of the project we sent interviewers into these geographic areas and women of childbearing age were interviewed to assess their knowledge of maternal and newborn health and utilization of skilled attendants at delivery. The results of these 1350 interviews constitute the data to be reported in this article. In one area, Eburru, we had completed training the number of CHWs that funding allowed, while in the other two areas, CHW training was continuing. The CHWs in Eburru were followed up for 1.5 years after these interviews were done in order to assess attrition of volunteers over time. Ongoing training in Nyakio and Kinale precluded a similar attrition assessment in those two regions.

### Core Content

The core content was a compilation of evidenced based practices in maternal and newborn health including the importance of prenatal care and having a birth plan, the value of delivery with a skilled attendant, danger signs in pregnancy, danger signs in the newborn, kangaroo care where skin to skin contact with the newborn promotes temperature control, the importance of exclusive breast feeding for 6 months, and maternal and infant nutrition. The fundamental role of the CHW in Kenya is in delivery of health prevention and promotion messages and to give appropriate referral, encouraging people to utilize the health system for further evaluation. The core content described above and the central functions of the CHWs map onto the curriculum developed by the Ministry of Health (MOH) and published in 2011 (after data collection was completed) titled, *Community Based Newborn and Maternal Care: A Training Course for Community Health Workers* [17]. The topics of the curriculum are listed in Table 1.

**Table 1:** Curriculum Topics.

Session #	Topics
1	Community engagement and interacting with families, roles and responsibility of CHW, Importance of home based maternal and newborn care, importance of a birth plan, ANC, facility delivery
2	Normal pregnancy, human reproductive physiology, danger signs in pregnancy, importance of ANC, facility delivery, know HIV status, tetanus immunization
3	Assessment of the newborn, danger signs in newborns, how to do referrals and home visits,
4	Immediate care of newborns, kangaroo care, cord care,
5	Breast feeding and general nutrition for pregnant woman and feeding of infants
6	Post pregnancy care, danger signs after delivery, family planning, data collection for CHWs and linkages with health system

### Timing and spacing of CHW training

A community based participatory process was used to refine the training of CHWs in the first two sites, Eburru and Nyakio. The spacing of training days was modified in the initial implementation period, due to feedback from participants. The training was delivered to groups of 10–15 people that met once to twice a month. Having a training schedule not on consecutive days was more suitable for the lifestyle needs of participants who were small scale farmers. This spaced approach to training was adopted in the hope that it would improve retention of volunteers. The training of CHWs utilized a mentored skill practice approach appropriate to adult learners who were of limited literacy. All CHW trainees were rated for competency prior to being allowed to graduate from the training. CHW trainees were blind to these competency assessments and trainees were supported with supervised skill practice until they were able to independently deliver the messages.

This training was ultimately rolled out in three geographically distinct areas that represented administrative areas (sub-locations). Community agreement and support was obtained prior to the start of the CHW training. The community leadership was involved with identification and approval of volunteers to ensure reliability of and future recognition of volunteers. The number of CHWs who were trained in each area was based on time and resources of the project during the three-year period prior to the study. The ratio of trained CHWs to number of households in the three geographic areas ranged from 1∶37 to 1∶820. None of the areas achieved 1∶20 ratio as recommended by the government [9]. However the range observed in this study is consistent with the operational constraints often experienced by the Kenya Community Health Strategy. In Eburru, the area where the ratio of CHW was 1∶37, the CHWs were followed after this data collection for 18 months to assess attrition rates. (Table 2).

**Table 2:** Density of CHWs to households.

Study-site	Time of exposure to the project	Number of trained CHWs	CHW ratio per household
Eburru	3 years (3 classes of about 15)	42	1 CHW per 37 households
Nyakio	3 years (2 classes of about 15 and anotherclass began just after study data collection)	34	1 CHW per 252 households
Kinale	18 months (one class of 7 completed and another class of 12had started at the time of data collection)	7	1 CHW per 820 households

### Study Design

The study utilized a quasi-experimental nonequivalent comparison group design (exposed to the CHW delivered health messages vs not exposed) that examined relevant demographic items and knowledge about maternal and newborn health combined with a comprehensive retrospective birth history of womańs children using oral interviews. This study included posttest only measures and exposure was self-reported by the participants in a personal interview done in the local language. In addition, CHWs in Eburru were followed 18 months after completion of training to assess attrition rates.

Interviews were conducted during a two-month time frame between August and September 2011. Women of childbearing age were invited to participate in interviews. A systematic random sampling method was utilized to obtain interviewees from a representative sample of households from each region and was based on a mapping of the regions done to minimize potential for sample bias. Interviewers commenced their work from a central point and field staff interviewed a woman in every third household. Where household density was very low, a convenience sample was obtained to ensure that all areas of the geographic region were included.

The interviews had five parts: (1) informed consent with inclusion and exclusion criteria; (2) demographics; (3) a retrospective review of information about previous pregnancies and deliveries including a question “Place where child was born: home, name of specific facility, or other?”; (4) knowledge questions about healthy pregnancy, delivery and neonatal care and (5) a question, “Did you attend a teaching of the “Kijabe Newborn Community Health project” to determine exposure to intervention. Knowledge, demographic, and birth history questions were adapted from questions in the Kenya Demographic Health Survey. Knowledge questions were selected based on material taught to the trainees and were vetted by a panel of experts in maternal and newborn care. Detailed knowledge questions on danger signs in the pregnant woman and newborn, newborn care and nutrition were comparable to questions asked in other validated tools [18]. (A copy of the questionnaire is available from the authors on request).

### Data Analysis

Data were entered into Excel and later transferred and analyzed with the Statistical Package for Social sciences (SPSS) version 20.0, and with R (version 2.14.1). Descriptive statistics were computed to summarize demographic characteristics of the respondents and frequencies of correct answers to knowledge questions were examined to estimate the impact of the intervention on knowledge of maternal and newborn health. The sum of correct answers across all knowledge questions was compared for those who did and did not report exposure to health messages from a CHW, and the knowledge mean score was computed by averaging all respondents’ test scores. The differences of the knowledge mean scores were evaluated using independent-samples t-test. The percentage of women exposed and not exposed to health messages and who chose to deliver in a facility with a skilled birth attendant was reported by year and analyzed using Pearson Chi Square.

## Results

1,398 women were approached for interviews the three regions where CHWs were trained. Forty-one women did not meet inclusion or exclusion criteria or refused to participate and an additional seven women withdrew after answering only a few questions. 1350 women completed interviews. Modal age was 26–30 years. About 80% of women in the sample reported being married. Economic and educational background varied by area with women in Eburru having the least education (7.38 years) and lowest economic level with 60% of families living in a temporary (mud and stick) house (See Table 3).

**Table 3:** Demographics of each site.

Region		Eburru N = 373		Kinale N = 484		Nyakio N = 482	
Exposure to teaching		No N = 272	Yes N = 101	No N = 379	Yes N = 105	No N = 295	Yes N = 187
Age	Mode age in years	26–30	26–30	26–30	36–40	31–35	31–35
Education	Mean number of years of school attendance	6.7*	7.6*	7.9	8.3	8.2	8.4
	No schooling	8%*	1%*	1%	2%	2%	1%
	Some primary	40%*	39%*	37%	29%	34%	32%
	Completed primary	43%*	47%*	41%	44%	37%	39%
	Some secondary or more	9%*	13%*	21%	25%	27%	28%
Economic status	Permanent house (Concrete Block)	4%*	2%*	11%	7%	22%	31%
	Semi-permanent house (wood frame)	42%*	23%*	86%	92%	71%	64%
	Temporary house (sticks and mud)	54%*	75%*	2%	1%	7%	5%
Family status	Married or living together	86%	83%	76%*	85%*	75%	85%
	Widowed	1%	2%	5%*	0%*	4%	3%
	Divorced or separate	3%	5%	6%*	8%*	8%	4%
	Single or never been married	9%	10%	13% *	7%*	13%	8%
Number of pregnancies	Mean number of carried pregnancies per woman	3.6*	4.1*	3.8	4.0	3.3*	3.7*
Age of the mother during first delivery	<18 years	37%	39%	33%	27%	25%	27%
	19–22 years	50%	48%	50%	53%	49%	50%
	23–30 years	13%	13%	16%	19%	25%	22%
	>31 years	0%	0%	1%	1%	1%	0%
Distance to next health facility by foot in time	<1 hour	44%	41%	70%	80%	86%	85%
	1–2 hours	23%	36%	29%	19%	13%	13%
	>2 hours	33%	22%	1.0%	1.0%	1%	2%
	Mode distance in minutes	60–90	60–90	30–60	15–30	30–60	30–60

*Denotes significant differences at the p<0.05 level using Chai Square or t-test to compare participants and non-participants.

When we examined the knowledge score within the 3 areas where CHWs were giving health messages, the mean knowledge score was higher among women who reported receiving one or more health messages, compared to those who did not: Eburru 32.3 versus 29.2 (t_(143)_ = −3.98, p<0.001); Kinale 21.8 vs 20.7 (t_(282)_ = −1.87, p<0.07); Nyakio 26.6 vs 23.8 (t_(136)_ = −4.88, p<0.001).

**Figure 1:**
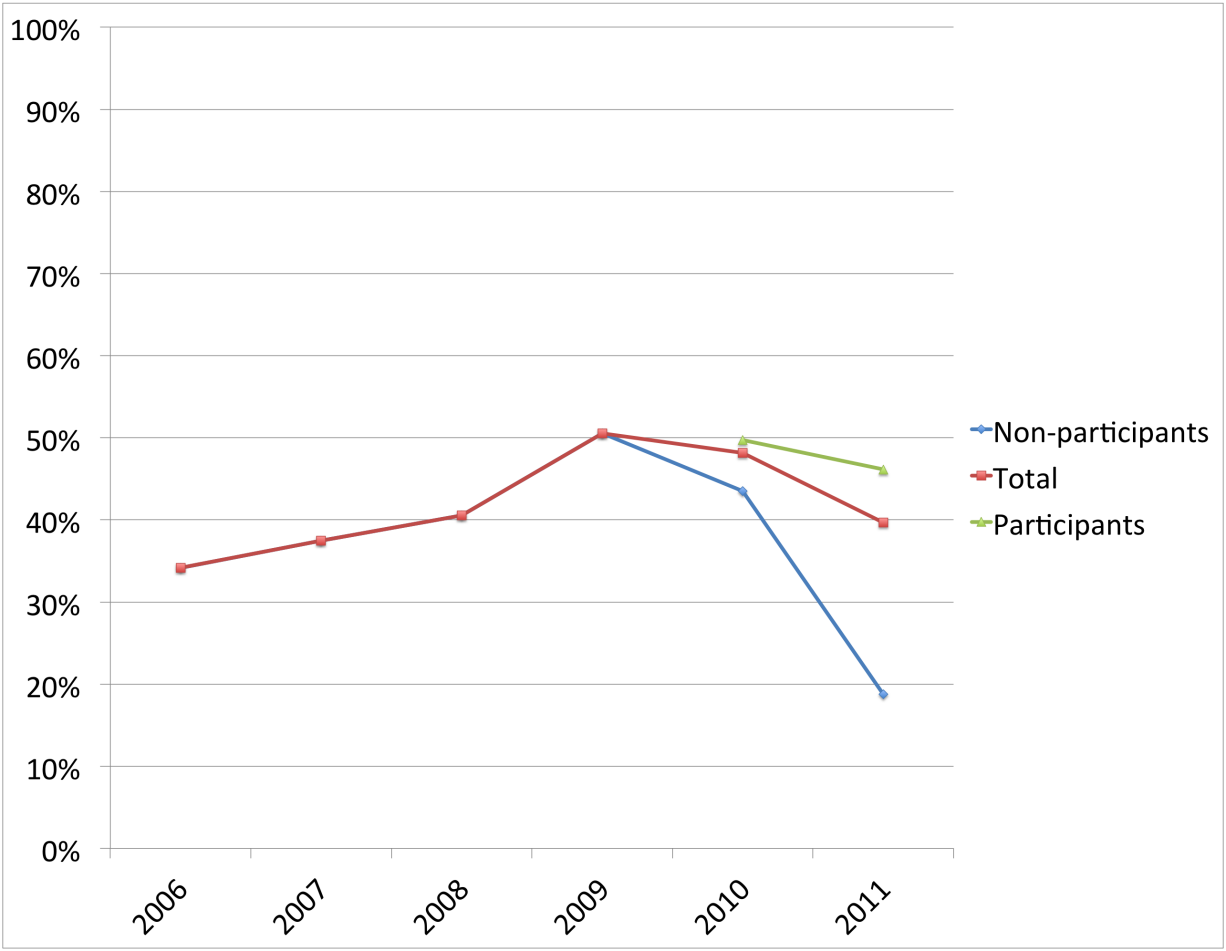
The percent of women delivering at a facility in Eburru, where the intervention began in 2009. The diverging lines after 2009 compare women who participated in the intervention with those who did not. (*X^2^* = 3.83 (1, N = 68); p = 0.05.

**Figure 2:**
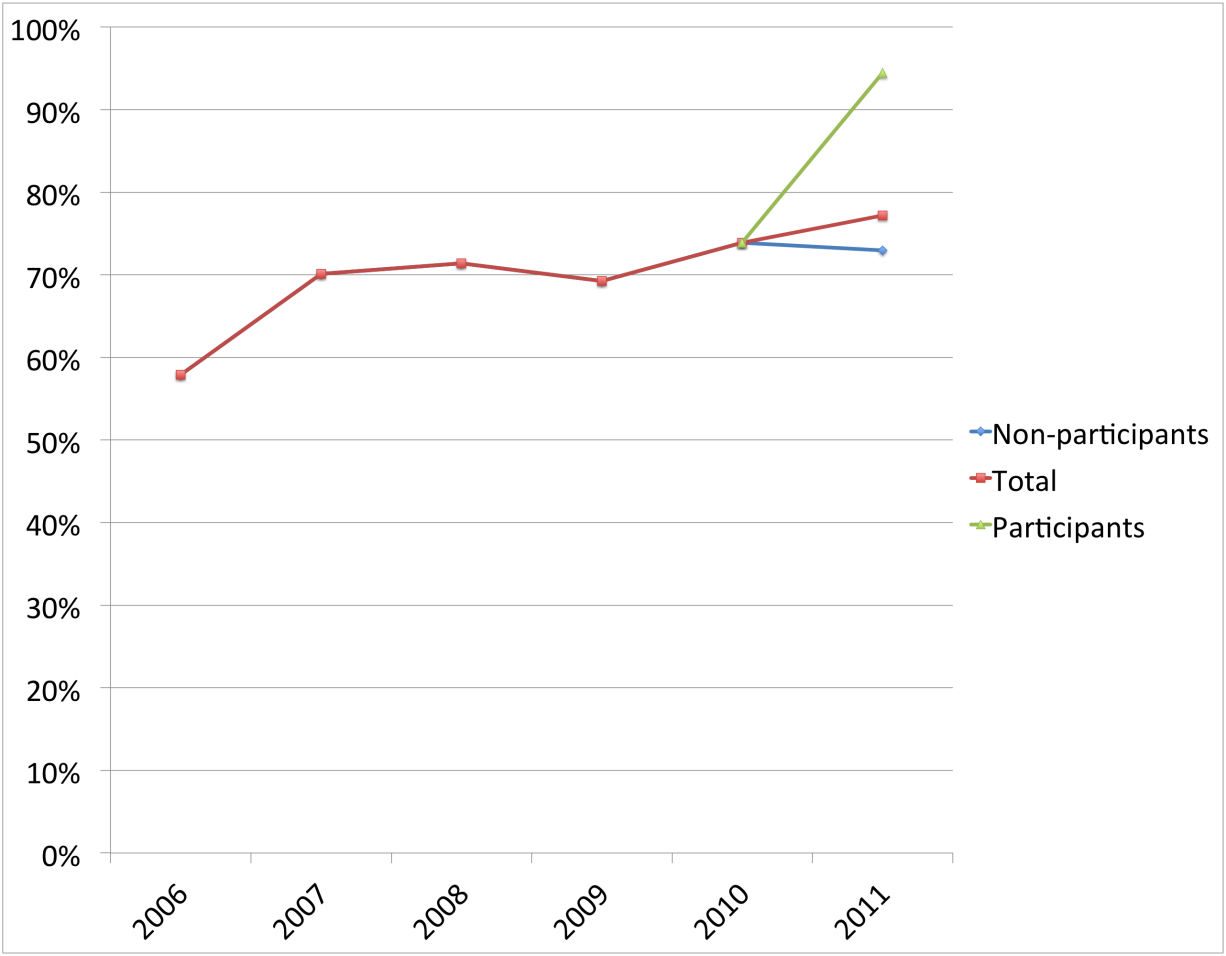
The percent of women delivering at a facility in Kinale, where the intervention began in 2010. The diverging lines after 2010 compare women who participated in the intervention with those who did not. (*X^2^* = 3.78 (1, N = 92); p = 0.06).

**Figure 3:**
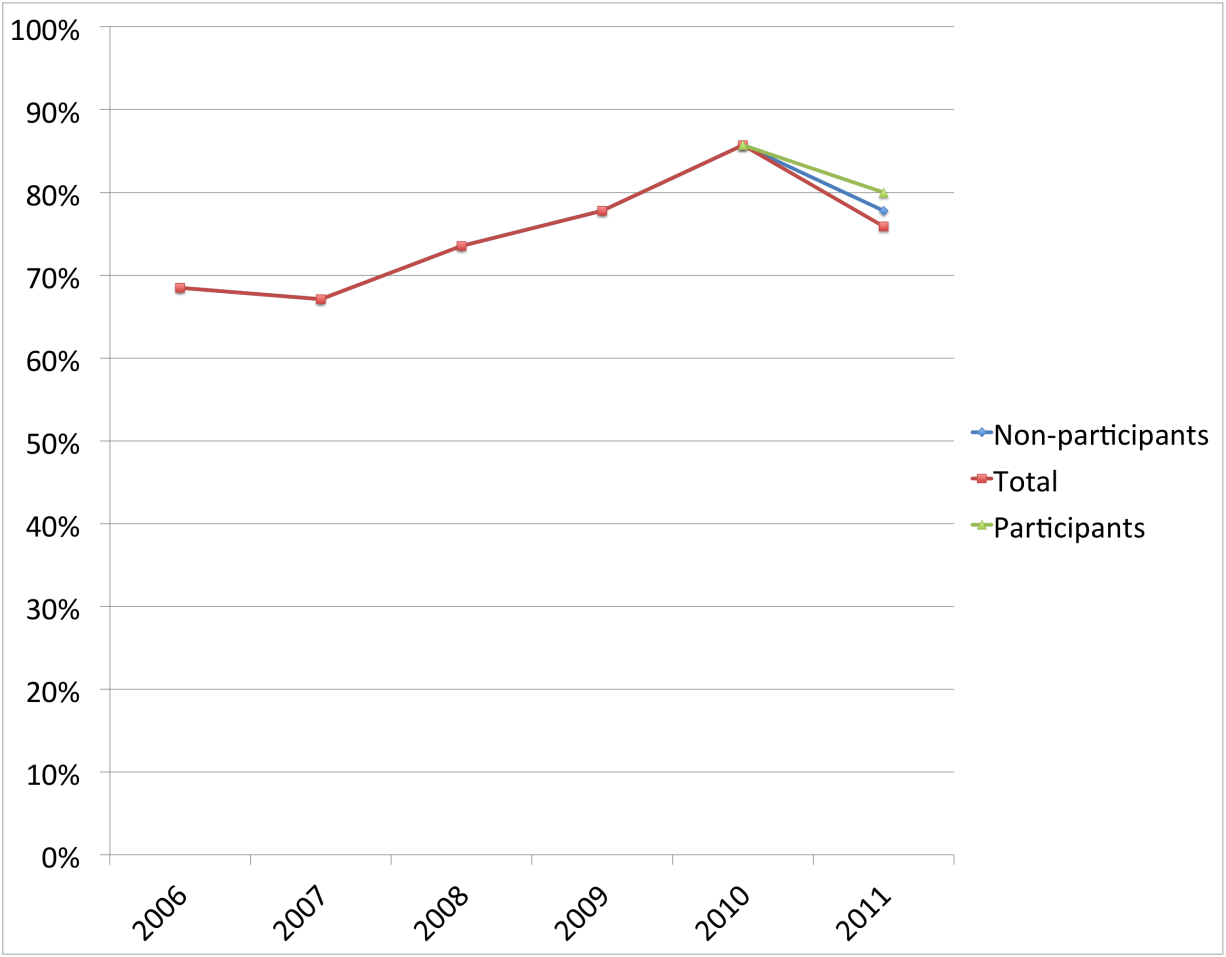
The percent of women delivering at a facility in Nyakio, where the intervention began in 2010. The diverging lines after 2010 compare women who participated in the intervention with those who did not. (*X^2^* = 0.44 (1, N = 83); p = 0.83).

In each of the three separate areas where CHWs were trained, the number of women delivering under skilled attendance was higher among those mothers who reported receiving at least one health message, compared to those who did not. The percentage of facility deliveries of women exposed to the CHW delivered health messages versus non exposed was; Eburru 46% versus 19% (*X^2^* = 3.83 (1, N = 68); p = 0.05); Kinale 94% versus 73% (*X^2^* = 3.78 (1, N = 92); p = 0.06): and Nyakio 80% versus 78% (*X^2^* = 0.44 (1, N = 83); p = 0.83). (See Figure 1–3).

Long term follow up data on retention rates of these volunteer CHWs in Eburru was examined 1.5 years after the training was completed in the region. Volunteer retention rates were greater than 80%. The project continued to work in the other geographic sites, training additional volunteers so retention rates are not yet available.

## Discussion

This study documents that a group of community volunteers who are primarily subsistence farmers in Kenya may effectively increase knowledge of maternal and newborn health in their communities and may promote delivery under skilled attendance. The AIC Kijabe Maternal Newborn Community Health (KMNCH) project functioned in three geographically separate areas, and in each area women exposed to the intervention had more knowledge of maternal and newborn health issues and were more likely to choose a facility delivery. Effect size was smallest in areas where women were of higher educational and economic status and where rates of deliveries were higher at baseline, but the effect was still in the hoped for direction.

The finding supports the Kenya policy to promote health through a direct person-to-person trust based spread of health messages. This finding may infer that trusted personal relationships form a part of the mechanism for knowledge to translate into behavior change.

The magnitude of the intervention impact seems to be influenced by local contextual factors. In areas where greater educational opportunity or greater economic resources were available, more women were choosing a facility-based delivery at baseline. In these more advantaged areas like Nyakio the positive influence of the intervention was present, even though the effect size was significantly smaller. The magnitude of the intervention impact may also have been influenced by the penetration of the messages based on the number of trained volunteer CHWs per number of households.

The intervention was also striking for the retention rates of the volunteers. Eighteen months after the KMNCH team finished training in Eburru, retention was more than 80%. These retention rates are higher than those reported in other Kenyan studies where drop out rates of 33%–40% were noted [15], [19]. The potential impact of curriculum timing and spacing on retention rates is an area ripe for future qualitative and quantitative research.

This study adds to the literature in several ways. It adds to the small but growing body of literature addressing the continuum of care from the community to the facility [20]. The primary objective this volunteer CHW project was to improve knowledge. The secondary goal was to drive demand for skilled delivery. The results are comparable to published data from Burkina Faso where a 14% increase in deliveries was noted [21].

This study adds support toward the validation of the core content packaged in the recently published training manual *Community Based Newborn and Maternal Care: A Training Course for Community Health Workers* [17]. Field-testing of this package of evidenced based interventions supports the assertion that volunteers can achieve improvements in health promotion and prevention while operating within the Kenyan Ministry of Health scope of work.

This study illustrates a potential role for community based participatory processes in intervention implementation. The innovations made to the spacing and interval of training delivery was a process that required community input, not input from professional educators. It is possible that the slower pace of training, one adapted to the agrarian lifestyle, contributed substantially to the retention of volunteers. The strong relational connections developed through the mentoring and skill practice were also elements driven by community needs. When working with adult learners, especially those with low literacy, skill practice under supervision may prove an effective motivational tool building on the strength of their oral and relational capacity.

This study demonstrates utilization of a low cost study design to gain a longitudinal perspective by combining a cross sectional approach with a retrospective birth history of all the children each woman had. Although repeated measures are able to offer a stronger inference of program effect, they are very labor intensive and expensive. In resource-limited settings the opportunity to examine evidence of effectiveness does not need to be eschewed due to cost.

Finally, this study is an effectiveness trial. Effectiveness studies are of special interest in resource constrained areas because of the ability to examine the program in a real world setting under actual conditions. Effectiveness trials using quasi-experimental designs do not address as many potential threats to validity as do randomized trials, but this is clearly a case where the good should not be an enemy of the best [22].

This study has some limitations. This intervention was adapted for rural agrarian populations and the improvements in women accessing a facility-based delivery may not be generalizable, especially to pastoralist populations. Self-report was utilized in the questionnaire. Self report especially where it requires increased length of time for recall is subject to bias. However, the birth and death of children are significant events and place of delivery is likely to be retained in memory. Messages delivered to those who reported exposure to the KMNCH was variable. The study design does not control for alternative explanations that could have been reasons for improvements. This study did not have high coverage of the project regions. No area achieved the government recommended ratio of CHWs to population. More substantial project coverage meaning a ratio of trained CHWs to population closer to the 1CHW: 20 households rate recommended by the government combined with increased capacity within the facilities may be required to demonstrate population level improvements in maternal and newborn mortality [23], [24]. But the ability to demonstrate improvements in proximal indicators such as knowledge and increased number of facility based deliveries does provide reassurance that processes are on track to achieve desired objectives when scale ups of interventions are ready.

## Conclusion

The KMNCH project demonstrates that community based participatory processes may be useful in developing appropriate contextual adaptations of evidenced based interventions and that volunteers from the community can effectively share health messages that increase knowledge of maternal and newborn care and encourage deliveries under skilled attendance with increases of 2–21% in three separate regions in Kenya. The study adds support toward validation of the core concepts contained in the *Community Based Newborn and Maternal Care: A Training Course for Community Health Workers* [17]. It identifies an approach where more than 80% of the CHWs continue to volunteer their time to promote maternal and newborn health and are actively working with district health teams 18 months after completion of training.
